# The Asian house shrew *Suncus murinus* as a reservoir and source of human outbreaks of plague in Madagascar

**DOI:** 10.1371/journal.pntd.0006072

**Published:** 2017-11-20

**Authors:** Soanandrasana Rahelinirina, Minoarisoa Rajerison, Sandra Telfer, Cyril Savin, Elisabeth Carniel, Jean-Marc Duplantier

**Affiliations:** 1 Plague Unit, WHO Collaborating Center, Institut Pasteur, Antananarivo, Madagascar; 2 School of Biological Sciences, University of Aberdeen, Aberdeen, United Kingdom; 3 Yersinia Research Unit and National Reference Laboratory, Institut Pasteur, Paris, France; 4 IRD, Centre de Biologie et de Gestion des populations (ird/inra/cirad/montpelliersupagro), Montpellier, France; University of California San Diego School of Medicine, UNITED STATES

## Abstract

Identifying key reservoirs for zoonoses is crucial for understanding variation in incidence. Plague re-emerged in Mahajanga, Madagascar in the 1990s but there has been no confirmed case since 1999. Here we combine ecological and genetic data, from during and after the epidemics, with experimental infections to examine the role of the shrew *Suncus murinus* in the plague epidemiological cycle. The predominance of *S*. *murinus* captures during the epidemics, their carriage of the flea vector and their infection with *Yersinia pestis* suggest they played an important role in the maintenance and transmission of plague. *S*. *murinus* exhibit a high but variable resistance to experimental *Y*. *pestis* infections, providing evidence of its ability to act as a maintenance host. Genetic analyses of the strains isolated from various hosts were consistent with two partially-linked transmission cycles, with plague persisting within the *S*. *murinus* population, occasionally spilling over into the rat and human populations. The recent isolation from a rat in Mahajanga of a *Y*. *pestis* strain genetically close to shrew strains obtained during the epidemics reinforces this hypothesis and suggests circulation of plague continues. The observed decline in *S*. *murinus* and *Xenopsylla cheopis* since the epidemics appears to have decreased the frequency of spillover events to the more susceptible rats, which act as a source of infection for humans. Although this may explain the lack of confirmed human cases in recent years, the current circulation of plague within the city highlights the continuing health threat.

## Author summary

The reemergence of plague is related to the persistence and dynamics of its reservoirs and vectors. During the human plague outbreaks that re-occurred in Mahajanga harbor, Madagascar in the 1990s, the shrew *Suncus murinus* was found infected with *Yersinia pestis*. Combining field surveys, experimental infections and genetic analysis, we examined the role of Asian shrew *Suncus murinus* in plague transmission and maintenance comparatively with others potential hosts in Mahajanga. The genomes of nineteen *Y*. *pestis* isolates recovered from humans, shrews, rats and fleas in this focus were sequenced and compared. We observed the predominance of *S*. *murinus* captured during the epidemics and their carriage of the flea vector. Shrews exhibited high resistance to *Y*. *pestis* experimental infections. Genetic analyses of the strains isolated from various hosts were consistent with two partially-linked transmission cycles, with plague persisting within the *S*. *murinus* population, occasionally spilling over into the rat and human populations. The isolation of a *Y*. *pestis* strain from a rat in Mahajanga in 2014 genetically close to shrew strains reinforces this hypothesis. The current circulation of plague within the city highlights the continuing health threat.

## Introduction

Plague, like other zoonoses, can exhibit large temporal variation in incidence at the same location, sometimes re-emerging after long periods of silence or apparently disappearing. Such variation in exposure may be due to changes in the presence or prevalence of the pathogen within the reservoir community or in the contact between humans and infected reservoirs (or vectors). Identifying key reservoirs of a zoonotic disease is essential for understanding disease dynamics, as well as for the design of surveillance or control strategies. However, long-term data from reservoir populations are relatively rare, especially in resource poor settings.

*Yersinia pestis*, the causative agent of plague, is usually transmitted to humans from rodents via the bite of infected fleas. Such bubonic plague cases can develop into pneumonic plague that may be transmitted human to human. Globally, most plague cases occur in Africa, with Madagascar, the country most seriously affected, reporting an average 400 human cases annually between 2010 and 2015 [1,2]. Plague arrived in Madagascar in 1898 at the port of Toamasina, subsequently spreading to other ports and then the Central Highlands from 1921. Plague became endemic in areas above 800 meters [3] but apparently disappeared from coastal areas after 1928. However, between August 1991 and April 1992, plague reappeared after more than 60 years of silence in Mahajanga, a city on the north-west coast of Madagascar with 202 suspected cases including 41 probable and confirmed human cases [4–6], after recolonization by a strain originating from the Central Highlands foci [7,8]. From 1995 to 1998, annual epidemics occurred with a total of 1702 suspected and 297 confirmed cases [9,10]. The last confirmed human case occurred in November 1999. Since then no new human cases were detected. From a public health viewpoint it is important to understand the role of different reservoir hosts in allowing plague to re-emerge in Mahajanga in the 1990s and whether plague persists today.

In rural areas of the Central Highlands in Madagascar, where most human cases occur, the epidemiology of plague is relatively well understood, primarily involving the black rat *Rattus rattus* and two flea vectors *Xenopsylla cheopis* and *Synopsyllus fonquerniei* [11,12]. Black rats in these areas have acquired resistance [13,14] and this may play an important role in persistence as the co-existence of resistant and susceptible hosts is often thought to play an essential role in the maintenance of plague [15].

In Mahajanga, plague epidemiology is poorly understood. The human plague season in this coastal focus is July to November while in Central Highlands foci it is September to April [16]. Preliminary studies found that the shrew *Suncus murinus* dominated the small mammal population with some infected by the plague bacillus and carriers of *X*. *cheopis* [6,17]. *S*. *murinus* is a small mammal belonging to the family *Soricidae*, order *Soricomorpha*. Originating from India, it is often commensal with man, living in and around houses, and has colonized South East Asia, the Pacific islands and the coasts of the Indian ocean [18,19] and was introduced to Madagascar in the 11^th^ to 14^th^ centuries [20,21]. The role of shrews as a reservoir of plague has been alluded to in Vietnam [22,23] but it remains hypothetical.

Integrating data from different approaches can significantly strengthen understanding of complex multihost systems. Using data and samples collected during and immediately after the epidemics in 1990s and recently (2011–2014), this study examines the role of *S*. *murinus* in maintaining the plague focus and as a source of the human epidemics by (i) comparing seasonal and inter-annual changes in reservoir host and flea abundance and seroprevalence of antibodies to plague amongst reservoir hosts; (ii) comparing sensitivity of current populations of potential reservoir hosts to two *Y*. *pestis* strains isolated from *R*. *norvegicus* and *S*. *murinus* during the epidemics; and (iii) conducting genetic analyses on whole genome sequences of *Y*. *pestis* isolates from Mahajanga.

## Methods

### Ethics statement

The study has been conducted in accordance with the Institut Pasteur guidelines (http://www.pasteur.fr/en/file/2626/download?token=YgOq4QW7) for animal husbandry and experiments. All experiments were performed at Biosafety level 2+ and verbal informed consent was obtained for sampling rats and shrews in the household.

### Sampling of small mammals and fleas

**Fig 1:**
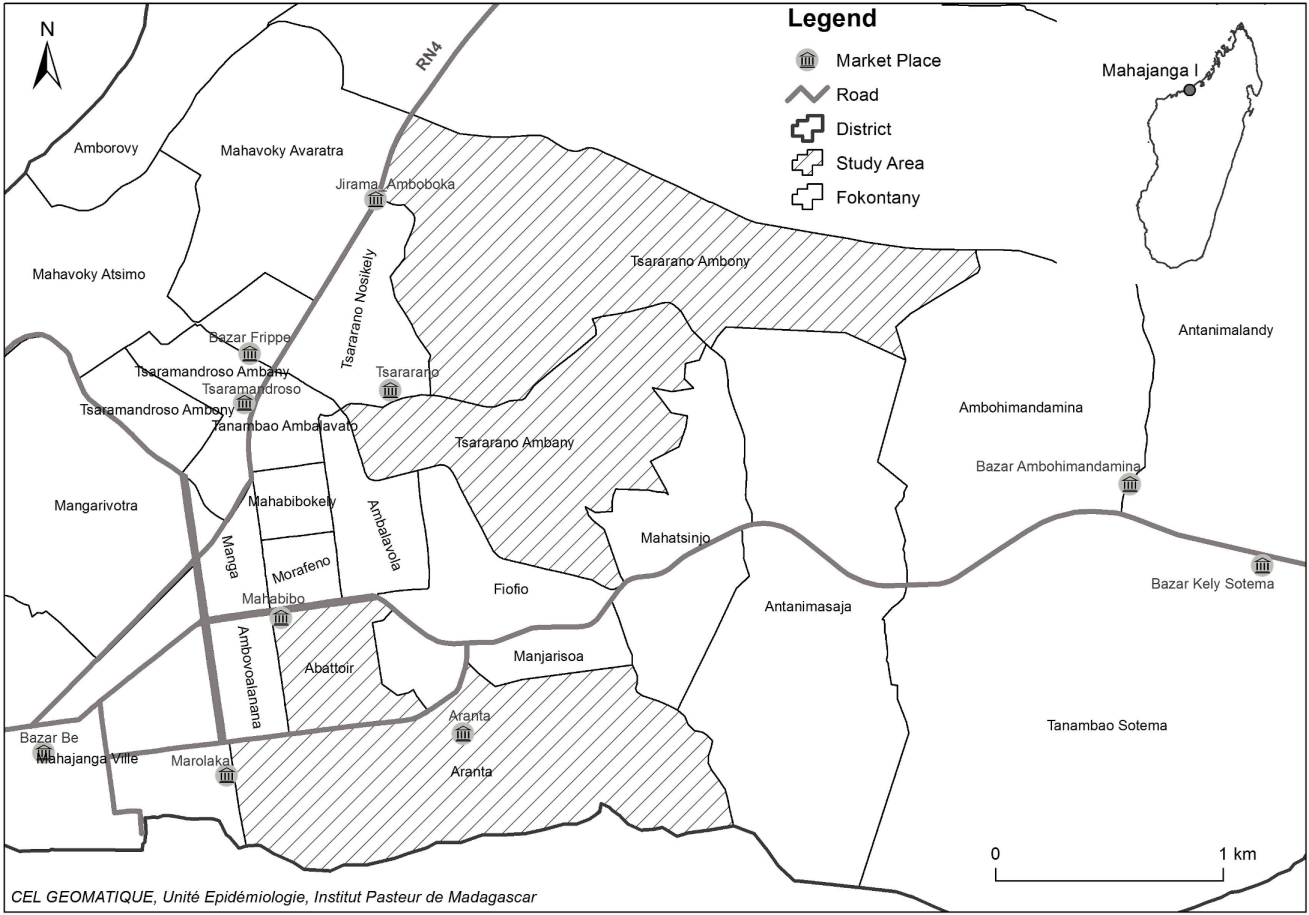
Map of Mahajanga city showing the location of the study areas. Data source: ArcGIS 10.4.1 for Desktop Public domain image: https://data.humdata.org/group/mdg and https://OpenStreetMap.org.

Trapping of small mammals was conducted in August 1991 and June to November 1995 in central Mahajanga (Abattoir and Aranta) (Fig 1), close to Marolaka market where the original re-emergence occurred in 1991 and where cases occured each epidemic season. From 1997–2001 and 2011–2014 datasets have been obtained with the same trapping regime (30 houses with one wire-mesh trap and one Sherman trap in each) in two areas: central and suburbs, Tsararano (Tsararano ambony and Tsararano ambany) that also had plague cases in 1995–1999 (Fig 1).

**Fig 2:**
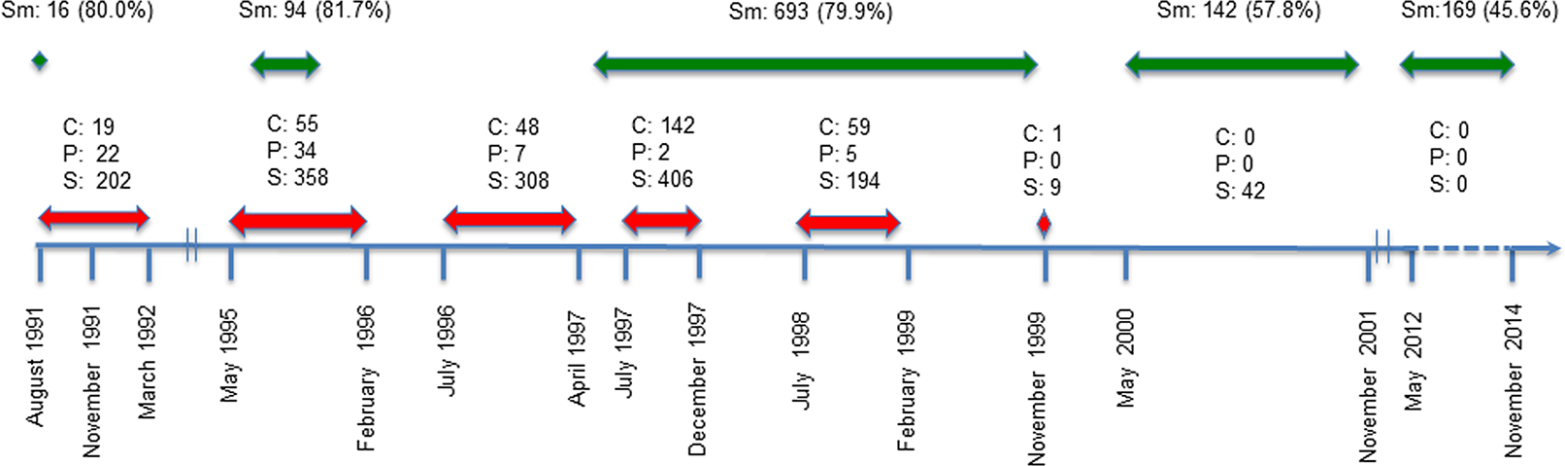
Chronogram of plague epidemics and animal trappings in Mahajanga: C: Number of human confirmed cases; P: Number of presumptive cases; S: Number of human suspected cases. Sm: no. of *S*. *murinus* trapped (% of total captures). Arrows red: human epidemic, green: rodent capture.

Traps were set for three consecutive nights either inside houses or in the immediate surroundings, baited with dried fish and onion each afternoon and checked every morning. Verbal informed consent was obtained for sampling rats in the household. As the last confirmed human case was November 1999, we classified trapping sessions into 3 periods: epidemic (quarterly from May 1997 to May 1999); post-epidemic (biannually from November 1999 to November 2001) and recent (biannually from November 2011 to November 2014). The Abattoir site was not sampled in November 2011. The periods of human plague outbreaks and animal trapping are schematized in Fig 2.

For each captured animal, fleas were collected. Proportion of animals infested and flea index (number of fleas per animal) were calculated for each host species-site-trapping session combination. Blood samples were collected on seropads (LDA 22440, Zoopole–Ploufragan). For dead animals, spleen samples were removed and stored in Cary Blair transport medium. In May and November 2012, live sub adult and adult individuals (weighing between 30 to 75g for shrews and 70 to 200g for rats) were brought to the laboratory for experimental infection. Blood samples were collected from the tail tip. Other captured animals were euthanized by cervical dislocation and necropsied.

### Plague diagnostics for wild animals

Two diagnostics tests were used: (i) culture on selective Cefsulodin Irgasan Novobiocin (CIN) agar for isolation of *Y*. *pestis* from spleen followed by identification with API 20E and phage lysis test [24,25]; (ii) enzyme-linked immunosorbent assay (ELISA) to detect specific anti-F1 IgG antibodies against *Y*. *pestis* in blood as previously described for mice and rats (with an optical density threshold of 0.05) [26–28]. For shrews, protein A-peroxidase diluted 1/10,000 was used as a secondary antibody, with a threshold of 0.1. Plates were read at 492 nm using Lab system Multiscan.

### Plague challenge experiment

Shrews and rats brought to the laboratory were quarantined for at least 15 days before the experiment and all animals used for infection were negative for anti-F1 antibody. During the experiment, animals were housed individually in cages with filtered enclosures at ambient room temperature with food and water *ad libitum*.

Two *Y*. *pestis* strains isolated during the 1998 epidemic in Mahajanga were used in the experiment: 163/98Sm, isolated from a *S*. *murinus* and 164/98Rn, isolated from a *R*. *norvegicus*. Virulence of the two strains was determined by measuring the lethality of OF1 mice infected subcutaneously with 100 colony-forming units (CFU) of *Y*. *pestis*. Based on previous studies on wild rats from Madagascar, two different doses of *Y*. *pestis* were used: 10^2^ CFU (LD_50_ previously reported for rats from the plague free zone in Madagascar) and 10^5^ CFU (LD_50_ for rats from the central highland plague focus) [13,14]. For each bacterial dose, 12 *S*. *murinus*, 10 *R*. *rattus* and 10 *R*. *norvegicus* were infected subcutaneously. The concentration of *Y*. *pestis* administered was estimated by measuring the optical density at 600 nm (LKB Biochrom, Ultrospec PLUS) and confirmed by colony enumeration of ten-fold dilutions on selective CIN agar plates. Animals were monitored twice daily for 21 days.

A rapid diagnostic test (RDT) was carried out on the spleen of dead rats to detect the presence of *Y*. *pestis* F1 antigen [29] in order to confirm that death was due to plague. Blood was collected on seropads at 0, 2, 4, 7, 14 and 21 days post infection to allow detection of anti-F1 IgG antibodies as described above. At the end of the experiment, rats were euthanized by CO_2_ asphyxiation and necropsied.

### Genetic relationships between *Y*. *pestis* strains

Whole genome sequencing of *Y*. *pestis* strains (Table 1) was performed using the NEXTflex PCR-Free DNA-Seq kit for Illumina (Bio Scientific), and a HiSeq2000 machine (Illumina, San Diego, CA) to yield paired-end reads of 100 bases. Image analysis, base calling, and error estimation were performed using Illumina Analysis Pipeline version 1.8. Paired-end Fastq files were uploaded into the *Yersinia* database available in the Enterobase website (http://enterobase.warwick.ac.uk/). De novo assembly of the genomes and SNP calling against the CO92 reference genome (after correction according to Morelli et al., 2010) [30] were performed automatically using EnteroTools in the Enterobase website.

**Table 1:** Epidemiological characteristics of the 19 strains of *Y*. *pestis* used for SNP typing.

Strain name	Isolation year	Location	Source	Origin
**110/95**	1995	Ambovoalanana	Human	Bubo
**120/95**	1995	Amboboka	Human	Bubo
**159/95**	1995	Ambalavola	*R*. *norvegicus*	Spleen
**170/95**	1995	Ambalavola	*X*. *cheopis* on *S*. *murinus*	Flea Homogenate
**171/95**	1995	Ambalavola	*X*. *cheopis* on *S*. *murinus*	Flea Homogenate
**175/95**	1995	Ambalavola	*R*. *norvegicus*	Spleen
**114/97**	1997	Antanimalandy	Human	Bubo
**265/97**	1997	Tanambao Sotema	Human	Bubo
**106/97**	1997	Tsararano ambony	*S*. *murinus*	Spleen
**103/97**	1997	Abattoir	*S*. *murinus*	Spleen
**89/95**	1995	Marolaka	*S*. *murinus*	Spleen
**94/98**	1998	Abattoir	*S*. *murinus*	Spleen
**93/98**	1998	Abattoir	*S*. *murinus*	Spleen
**89/98**	1998	Aranta	*X*. *cheopis* on *S*. *murinus*	Flea Homogenate
**169/98**	1998	Abattoir	*R*. *norvegicus*	Spleen
**163/98**	1998	Abattoir	*S*. *murinus*	Spleen
**118/98**	1998	Aranta	*X*. *cheopis* on *R*. *norvegicus*	Flea Homogenate
**98/98**	1998	Aranta	Human	Bubo
**89/14**	2014	Marolaka	*R*. *norvegicus*	Spleen

### Statistical analysis

Generalized linear models (GLM) were used to examine changes in the abundance of *S*. *murinus*, *R*. *norvegicus* and fleas (poisson errors and a log link) and the proportion of animals carrying fleas (binomial errors and a logit link). For models of flea abundance, the natural log of the number of host was used as an offset (these models analyze the flea index described above). Explanatory variables included month, spatial area and a variable to examine long-term changes. Two global models were considered, one considered a linear time trend, whilst the second included time period (epidemic, post-epidemic, recent). Two-way interactions between site and month and site and the long-term change variable were included. Initial choice between global models was based on Akaike Information Criterion (AIC) [31]. Where there was evidence of overdispersion we subsequently used quasipoisson, negative binomial or quasibinomial models, with model selection was based on the F test for “quasi” models and AIC for negative binomial models, with the most parsimonious model within 2 of the model with the lowest AIC selected. Due to lower power, seroprevalence data were analyzed using chi-square tests. To remove any effects of season, tests of long-term changes only used May and November data. All analyses were performed using R software [32].

## Results

### Small mammal and flea monitoring

Four species of small mammals were caught during trapping: *S*. *murinus*, *R*. *norvegicus*, *Mus musculus* and *R*. *rattus* (Table 2). *S*. *murinus* was systematically the most abundant small mammal captured over the entire study period followed by *R*. *norvegicus*. *R*. *rattus* were rare in 1991 and 1995 and then absent from the central areas. *M*. *musculus* were rare all along the study, except a slight increase recently in suburbs. All fleas collected were *X*. *cheopis* and fleas were significantly more abundant on *R*. *norvegicus* than *S*. *murinus* (p<0.001) (S1 Table).

**Table 2:** Comparison of the number of animals trapped in central areas in 1991 and 1995, and in both the central and peripheral areas from 1997 to 2014 in Mahajanga.

		1991		1995		Period epidemics (May 97—May 99)		Post epidemic (Nov 99—Nov 01)		Recent (Nov 11—Nov 14)	
	Species	No. trapped	%	No. trapped	%	No. trapped	%	No. trapped	%	No. trapped	%
**Central areas**	*M*. *musculus*	0	0	3	2,6	20	4.5	13	8.4	14	5.8
	*R*. *norvegicus*	2	10	15	13	49	10.9	59	38.3	129	53.1
	*R*. *rattus*	2	10	3	2,6	0	0.0	0	0.0	0	0.0
	*S*. *murinus*	16	80	94	81,7	379	84.6	82	53.2	100	41.2
	**Total**	20	100	115	100	448	100.0	154	100.0	243	100.0
**Suburbs**	*M*. *musculus*	NA	NA	NA	NA	14	3.8	11	7.7	23	13.0
	*R*. *norvegicus*	NA	NA	NA	NA	38	10.3	17	11.9	45	25.4
	*R*. *rattus*	NA	NA	NA	NA	41	11.1	16	11.2	17	9.6
	*S*. *murinus*	NA	NA	NA	NA	275	74.7	99	69.2	92	52.0
	**Total**	NA	NA	NA	NA	368	100.0	143	100.0	177	100.0

GLM analyses of host and flea abundance indicated significant long-term, as well as seasonal, changes, whilst site differences were limited to a moderate increase in flea abundance on *S*. *murinus* in suburbs (Table 2, S2 Table).

**Fig 3:**
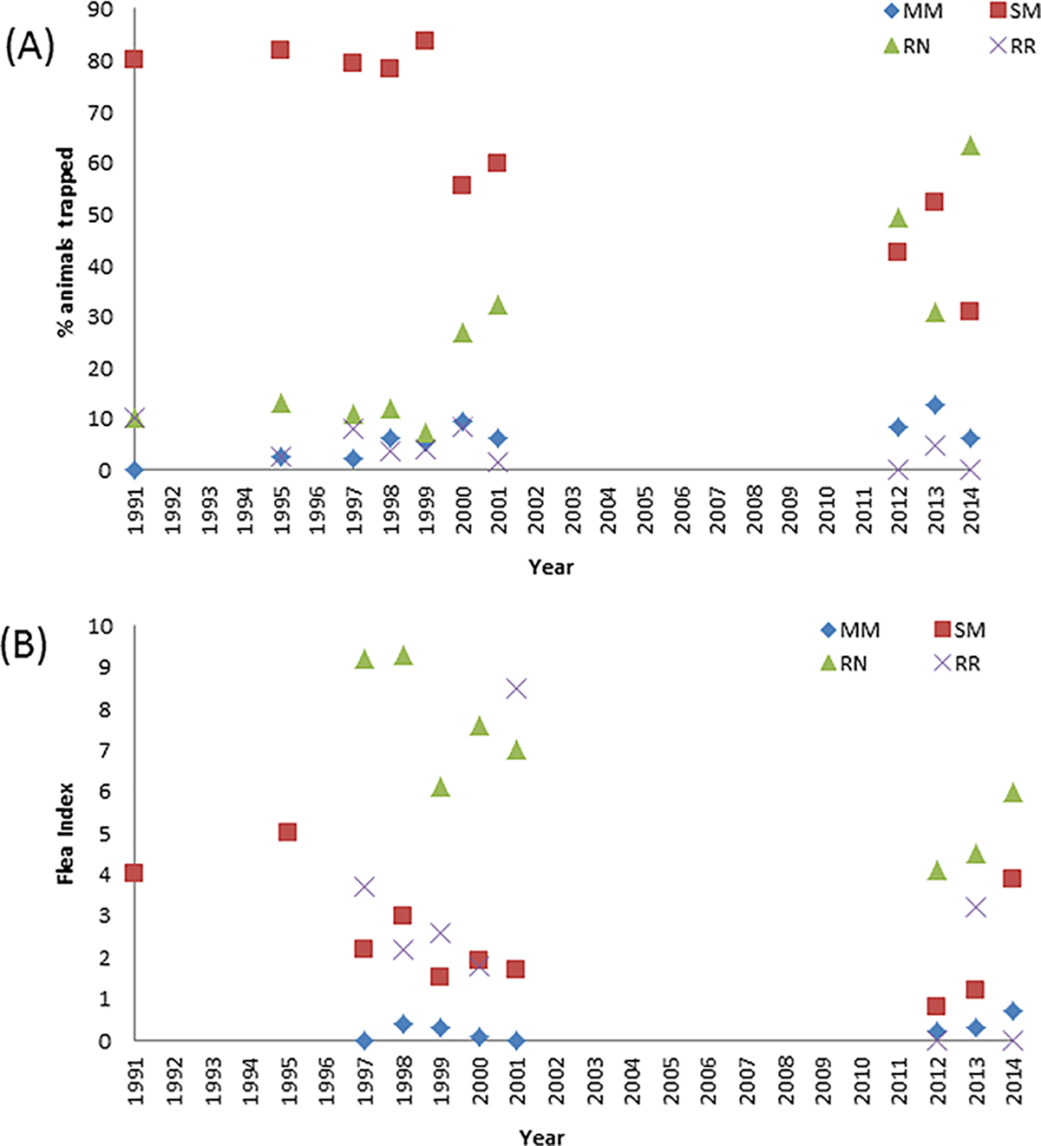
**Graphs showing: (A) Proportion of each mammal captured; (B) Flea Index of each mammal captured during the various trapping periods.** MM: *M*. *musculus;* SM: *S*. *murinus;* RN: *R*. *norvegicus*; RR: *R*. *rattus*.

For *S*. *murinus* abundance, comparisons of the initial two global models indicated that time period (AIC = 318.08) provided a better fit to changes in *S*. *murinus* abundance than a linear trend (AIC = 341.87). For all other datasets, a linear trend was the most parsimonious model. Relative abundance of *S*. *murinus* abundance was significantly lower in both the post-epidemic and recent periods, compared with the epidemic period, whilst *R*. *norvegicus* has shown a moderate increase in abundance over time (Fig 3A,S3 Table). *S*. *murinus* abundance appears to peak in May, whilst *R*. *norvegicus* does not show strong seasonality (S3 Table). For *S*. *murinus*, both the proportion infested with fleas and flea index showed a decline over time, whilst there was no evidence of long-term changes in flea abundance on *R*. *norvegicus* (S2 Table). However, flea index of *S*. *murinus* and *R*. *norvegicus* have tended to increase in 2014 (Fig 3B). In terms of seasonality, the flea index of *R*. *norvegicus*, the proportion of *S*. *murinus* carrying fleas and the *S*. *murinus* flea index peaked in August (S3 Table).

### Bacteriological and serological analyses of small mammals

Sixteen strains of *Y*. *pestis* strains were isolated during human plague epidemics (1991, 1995, 1997, 1998 and 1999) from small mammals: 9 from *S*. *murinus*, 6 from *R*. *norvegicus* and 1 from *R*. *rattus* (S1 Table). In 1998, fleas collected from 2 *R*. *norvegicus* and 1 *S*. *murinus* were infected with *Y*. *pestis*. In 2014, 15 years after the last confirmed human case, one strain was isolated from *R*. *norvegicus* caught close to the market of Marolaka, the place where the plague outbreak started in 1991.

Among seropositive animals, we have found that antibodies against plague were detectable in shrews throughout the entire period of study. Seroprevalence in *S*. *murinus* was higher in May (12.1%, 55/456) and August (12.0%, 15/125), than November (6.3%, 18/284) and February (2.6%, 3/114) (χ^2^_3_ = 12.76, p<0.01). There was no evidence that seroprevalence in *S*. *murinus* changed between time periods. In contrast, seroprevalence in *R*. *norvegicus* was higher during the epidemic period (16.1%, 13/81) than in the post-epidemic (0%, 0/75) or recent periods (2.3%, 4/172) (Fisher’s test: p<0.001) (S1 Table).

### Plague challenge experiments

Upon subcutaneous infection of OF1 mice with 100 CFU of either 163/98Sm (isolated from *S*. *murinus)* or 164/98Rn (isolated from *R*. *norvegicus*), no significant differences in terms of lethality were observed between the two strains: 6/6 and 5/6 mice died after infection with 164/98Rn and 163/98Sm, respectively.

**Fig 4:**
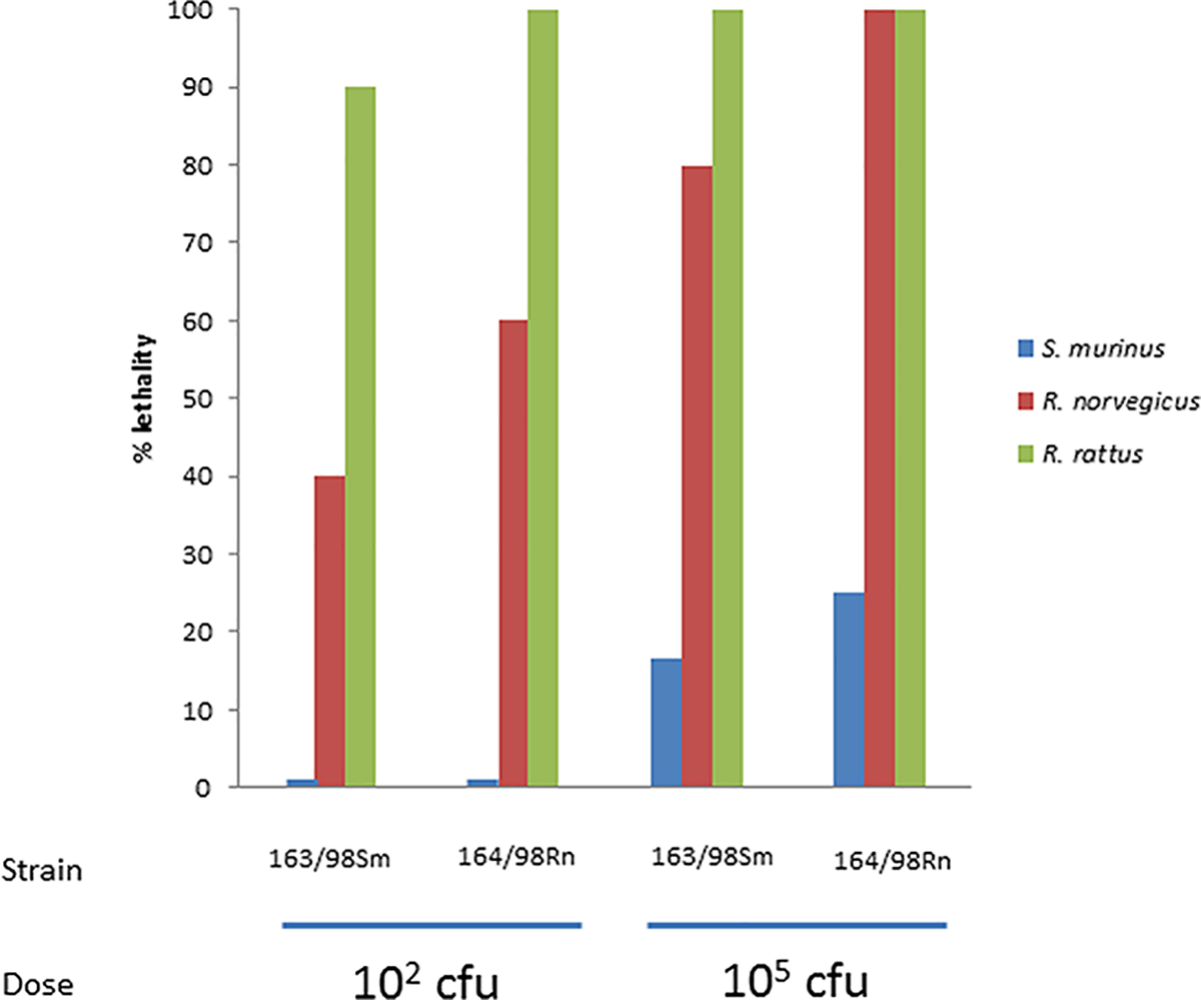
Percentage of lethality across the three mammal species during plague challenge experiments.

*S*. *murinus* was by far the most resistant species since no animals died after infection with 100 CFU, and most animals (≥75%) survived an infection with a high dose of 10^5^ CFU. *R*. *rattus* was highly susceptible to *Y*. *pestis*, even at a low dose of 100 CFU, while *R*. *norvegicus* exhibited intermediate levels of resistance (Fig 4). No major difference in lethality caused by the two strains was observed (p = 0.706).

Anti F1 antibodies were detected in both shrews and rats from day 7 following the infection.

### Genetic relationships between *Y*. *pestis* strains

**Fig 5:**
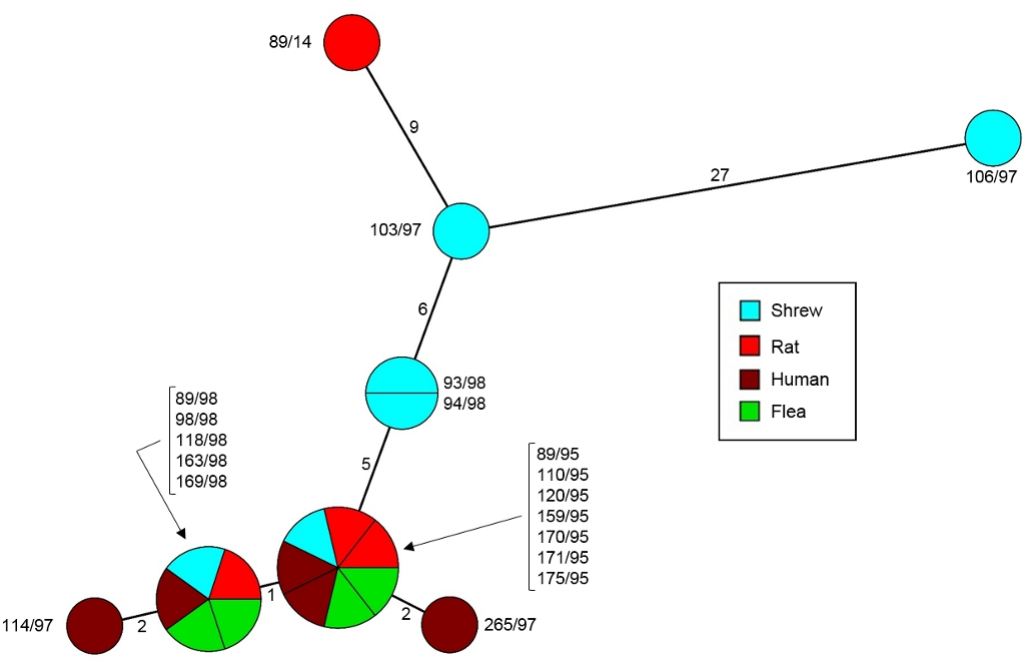
Minimum spanning tree of 4,098 SNPs for 19 *Y*. *pestis* isolates from Mahajanga. The origins of the strains are distinguished by colors. Strain name is mentioned close to the circle. Numbers on the branch reflect the number of SNPs between two circles.

To determine whether there was a possible exchange of strains between shrews and other infected hosts and vectors (humans, fleas and rats) during plague epidemics in Mahajanga, we evaluated their genetic relatedness. A SNP analysis was performed on the genomes of 6 *Y*. *pestis* strains isolated from *S*. *murinus* and 12 strains isolated during the same periods from humans, fleas and rats (Table 1). The resulting minimum spanning tree shows that the 7 *Y*. *pestis* strains from the 1995 epidemic were identical to each other, although they were isolated from different hosts (Fig 5). Five of the 7 strains isolated in 1998 from rats, shrews, fleas and humans were also identical, and they differed by a single SNP from the strains of the 1995 outbreak. However, 4 strains isolated from shrews in 1997–1998 differed from the others by a much higher number of SNPs and were also more distant among themselves (6 to 33 SNPs) (Fig 5). Since SNPs accumulate with bacterial divisions, this may be indicative of a very active multiplication of *Y*. *pestis* in shrews or a longer persistence in this animal population, leading to a higher bacterial diversification.

The *Y*. *pestis* strain (89/14) isolated from a rat in Mahajanga in 2014 branched with the separate shrew branch and was most closely related to a shrew strain (103/97), although this strain was isolated 17 years earlier (Fig 5).

## Discussion

Our study investigated the seasonal abundance of reservoirs and vectors for plague in Mahajanga (the only coastal foci in Madagascar), the dynamics of the antibody response in shrews and rats, the susceptibility of shrews to plague and the genetic relationship between *Y*. *pestis* strains isolated from humans, rats, shrews and fleas.

Integrating data from the different approaches used in our study provide strong evidence that *S*. *murinus*, a non-rodent host, was the most important reservoir of plague in the port of Mahajanga during epidemics in the 1990s, and since that date may have functioned as the maintenance host for plague within the city, despite a lack of confirmed human cases.

A reservoir can be defined as one or more epidemiologically connected populations which together achieve the critical community size and can, therefore, permanently maintain a pathogen and transmit the infection to the defined target population [33,34]. Thus, a species should be regarded as an important reservoir of plague if it is essential to maintenance of *Y*. *pestis* within a community of small mammals and/or if it acts as a source of infective fleas that pose a risk to humans. The predominance of *S*. *murinus* in traps during the epidemics in Mahajanga, their carriage of known flea vectors, and the isolation of *Y*. *pestis* strains from some of them suggest they played an important role in plague transmission and maintenance. Moreover, the close genetic relatedness among *Y*. *pestis* strains isolated from humans, the usual hosts and vector (rats, fleas) and from shrews, is indicative of an active circulation of *Y*. *pestis* strains between all these sympatric populations during outbreaks.

Despite the lack of human confirmed cases, the isolation from a rat in 2014 of a *Y*. *pestis* strain closely related to a strain isolated from a shrew in 1997 and the lack of long-term change in seroprevalence in shrews strongly argue for the maintenance of the plague bacillus in the shrew population over nearly 20 years. The findings from the experimental infection experiment are consistent with the ability of *S*. *murinus* to act as a maintenance host. The co-existence of susceptible and resistant hosts is thought to be important for persistence of plague within endemic foci [15,35], with susceptible hosts developing the high bacteremia needed to reliably infect fleas [36,37], and resistant hosts allowing the long-term maintenance of host and flea populations. In the desert plague foci of Central Asia, the great gerbil, with its high but variable rates of resistance, is thought to be the primary host [35,38]. Our results demonstrate that *S*. *murinus* exhibits a similar pattern, with high resistance overall but heterogeneous responses to infection at the individual level. Alternatively, some authors have suggested that individual hosts that survive infection could become chronically infected [39], potentially acting as a source of infection at a later date, possibly when declines in immunocompetence associated with ageing or coinfections reduce the ability of the host to control the infection. However, evidence for this is limited, and in laboratory experiments such chronic infections were caused by isolates lacking the ability to express the F1 antigen on their surface [39], an antigen that prevents phagocytosis of infected macrophages [40] and is thought to be essential for achieving the high bacteraemia necessary for transmission to fleas [41]. Thus, we believe heterogeneous responses, evidenced by our plague challenge experiments, in *S*. *murinus* are a more likely explanation. The presence of genetically divergent strains of *Y*. *pestis* from shrews provide additional evidence that plague persists within *S*. *murinus*, but occasionally spills over into the rat and human populations through partially-linked transmission cycles.

The seasonal patterns of host-vector abundance, seroprevalence and human cases are also consistent with the spillover of infection from *S*. *murinus*. Host abundance above a threshold has been shown to be critical for plague epizootics in other systems, such as within great gerbil (*Rhombomys opimus*) populations in Central Asia [42,43].

In Mahajanga, the increase in *S*. *murinus* abundance around May appears to lead to increased transmission (*S*. *murinus* seroprevalence peaks in May and August); whilst the increase in *X*. *cheopis* in August-November, when *S*. *murinus* have declined, suggest that this is the period that infected fleas are most likely to be seeking other hosts (rats), with the higher sensitivity of rats leading to more infected fleas that pose a risk to humans. In the Central Highlands in Madagascar, the start of the plague season in humans also coincides with a peak in flea abundance and a trough in host abundance [11]. Altogether then, our data point at shrews as a plague reservoir maintenance host, and suggest that this population may be at the origin of occasional resurgence of plague in Mahajanga. Although epidemiological data from Vietnam has previously suggested they may play a role in plague transmission along with rats [22,23], in Mahajanga they appear to be critical for persistence, with rats primarily acting as a “source population” for human infections. A similar epidemiological cycle of a relatively resistant reservoir allowing plague persistence and a second species facilitating transmission to humans was proposed for Java [44]. To fully understand the role of *S*. *murinus* in persistence, experiments are needed to establish the relative ability of susceptible and resistant individuals to infect fleas.

Our results also indicate that changes in the host-vector community may explain the lack of seasonal outbreaks in human cases since 1999, with a significant decrease in *S*. *murinus* abundance that appeared to primarily occur just after the outbreaks in the 1990s. The observed decline in *S*. *murinus* and *X*. *cheopis* since the epidemics appears to have decreased the frequency of spillover events to the more susceptible rats, which act as a source of infection for humans. However, based on seroprevalence data, this has not reduced plague transmission within *S*. *murinus* populations but the significant decline in seroprevalence in *R*. *norvegicus* after the outbreaks in humans.

The lesser abundance of *R*. *rattus* can be explained by a lower resistance to *Y*. *pestis* but also by competition with the larger *R*. *norvegicus* (the sewer rat) strongly favored by urbanization of the central districts. So today, *R*. *rattus* is only present in the suburbs with an important tree and shrub cover, a habitat more suitable for this arboreal species. However, they live in very close proximity to humans. In the period post epidemics, the abundance of *R*. *norvegicus* increases while *S*. *murinus* decreases: this suggest also competition between these two species, sharing the same subterranean and moist habitat and living greatly on the same food resources.

*X*. *cheopis* is the only effective vector in Mahajanga, consistent with evidence that the endemic flea *S*. *fonquerniei* is limited to the Central Highlands [16]. Although an experimental study indicated that *X*. *cheopis* live longer at higher relative humidity [45], the abundance of fleas on shrews presents a marked increase in August, the driest month in Mahajanga. This may provide an explanation for the discrepancy observed in plague seasons between Mahajanga and the main central foci in Madagascar. The optimal relative humidity for fleas may not occur at the same time in the two areas.

In conclusion, our results indicate that *S*. *murinus* acts as a maintenance host for *Y*. *pestis* in Mahajanga as well as a source of infection for other hosts. Although changes in the abundance of reservoirs since 1999 may have decreased the probability of infections in the human population, the confirmation that plague still circulates within the city and can spillover into other, more sensitive hosts highlights the continuing health threat. Given the spread of *S*. *murinus* to other countries, especially in port cities, they could also act as important reservoirs if plague is inadvertently introduced elsewhere. Our study also emphasizes the importance of collecting longitudinal data and integrating information from different approaches to elucidate the relative role of potential reservoir hosts of zoonoses.

## Supporting information

S1 Table Plague indicators during epidemics, post epidemics periods, and 15 years after the last human outbreak. (PDF)

S2 Table Final generalized linear models (GLM) models for analyses of small mammal and flea abundances. (PDF)

S3 Table Coefficients from final GLM models for small mammal and flea abundance. (PDF)

## References

[1] World Health Organization. Plague around the world, 2010–2015. Wkly Epidemiol Rec. 2016; 8: 89–104.

[2] World Health Organization (WHO). Plague in Madagascar: overview of the 2014–2015 epidemic season. Wkly Epidemiol Rec. 2015; 20: 250–252. 25980039

[3] Brygoo ER. Epidémiologie de la peste à Madagascar. Arch Inst Pasteur Madagascar. 1966; 35.

[4] Rasolomaharo M, Rasoamanana B, Andrianirina Z, Buchy P, Rakotoarimanana N, Chanteau S. Plague in Majunga, Madagascar. Lancet. 1995; 346(8984): 1234 7475693 10.1016/s0140-6736(95)92944-4

[5] Boisier P, Rasolomaharo M, Ranaivoson G, Rasoamanana B, Rakoto L, Andrianirina Z, et al Urban epidemic of bubonic plague in Majunga, Madagascar: epidemiological aspects. Trop Med Int Health. 1997; 2(5): 422–427. 9217697

[6] Laventure S, Rasoamanana B, Boisier P, Rasolomaharo M, Rahalison L, Randriantsoa J, et al Epidémies de peste urbaine à Majunga, côte ouest de Madagascar. Bull Soc Pathol Exot. 1998; 91: 85–6.

[7] Vogler AJ, Chan F, Nottingham R, Andersen G, Drees K, Beckstrom-Sternberg SM, et al A decade of plague in Mahajanga, Madagascar: insights into the global maritime spread of pandemic plague. MBio. 2013; 4(1). 10.1128/mBio.00623-12 PMC3573667 23404402

[8] Vogler AJ, Chan F, Wagner DM, Roumagnac P, Lee J, Nera R, et al Phylogeography and Molecular Epidemiology of Yersinia pestis in Madagascar. Plos Negl Trop Dis. 2011; 5(9).e1319 doi: 10.1371/journal.pntd.0001319 21931876 10.1371/journal.pntd.0001319 PMC3172189

[9] Migliani R, Ratsitorahina M, Rahalison L, Rabarijaona L, Rasolomaharo M, Razafimahefa M, et al La peste dans le port de Mahajanga: 6 habitants sur 1000 porteurs d ‘ anticorps anti-F1 en 1999. Arch Inst Pasteur Madagascar. 2000; 66: 6–8. 12463025

[10] Boisier P, Rahalison L, Rasolomaharo M, Ratsitorahina M, Mahafaly, Razafimahefa M, et al Epidemiologic features of four successive annual outbreaks of bubonic plague in Mahajanga, Madagascar. Emerg Infect Dis. 2002; 8(3): 311–316. doi: 10.3201/eid0803.010250 11927030 10.3201/eid0803.010250 PMC2732468

[11] Rahelinirina S, Duplantier JM, Ratovonjato J, Ramilijaona O, Ratsimba M, Rahalison L. Study on the movement of Rattus rattus and evaluation of the plague dispersion in Madagascar. Vector Borne Zoonotic Dis. 2009; 10(1): 77–84. 10.1089/vbz.2009.0019 20158335

[12] Andrianaivoarimanana V, Kreppel K, Elissa N, Duplantier JM, Carniel E, Rajerison M, Jambou R. Understanding the persistence of plague foci in Madagascar. PLoS Negl Trop Dis. 2013; 7: e2382 doi: 10.1371/journal.pntd.0002382 24244760 10.1371/journal.pntd.0002382 PMC3820717

[13] Rahalison L, Ranjalahy M, Duplantier JM, Duchemin JB, Ravelosaona J, Ratsifasoamanana L, et al Susceptibility to plague of the rodents in Antananarivo, Madagascar. Adv Exp Med Biol. 2003; 529: 439–442. doi: 10.1007/0-306-48416-1_87 12756805 10.1007/0-306-48416-1_87

[14] Tollenaere C, Rahalison L, Ranjalahy M, Duplantier JM, Rahelinirina S, Telfer S, et al Susceptibility to Yersinia pestis experimental infection in wild rattus rattus, reservoir of plague in Madagascar. Ecohealth. 2010; 7(2): 242–247 doi: 10.1007/s10393-010-0312-3 20443044 10.1007/s10393-010-0312-3

[15] Gascuel F, Choisy M, Duplantier JM, Debarre F, Brouat C. Host Resistance, Population Structure and the Long- Term Persistence of Bubonic Plague: Contributions of a Modelling Approach in the Malagasy Focus. PLoS Comput Biol. 2013; 9(5). 10.1371/journal.pcbi.1003039 PMC3649974 23675291

[16] Chanteau S, Ratsifasoamanana L, Rasoamanana B, Rahalison L, Randrianbelosoa J, Roux J, et al Plague, a reemerging disease in Madagascar, Emerg Infect Dis. 1998; 4: 101–104. doi: 10.3201/eid0401.980114 9452403 10.3201/eid0401.980114 PMC2627662

[17] Duplantier JM, Duchemin JB, Chanteau S, Carniel E. From the recent lessons of the Malagasy foci towards a global understanding of the factors involved in plague reemergence. Vet Res. 2005; 36(3): 437–453. doi: 10.1051/vetres:2005007 15845233 10.1051/vetres:2005007

[18] Yosida TH. Cytogenetical studies on Insectivora. II. Geographical variation of chromosomes in the house shrew, Suncus murinus (Soricidae), in East, Southeast and Southwest Asia, with a note on the karyotype evolution and distributions. Japanese J Genet. 1982; (1379): 101–111.

[19] Ruedi M, Courvoisier C, Vogel P, Catzeflis FM. Genetic differentiation and zoogeography of Asian Suncus murinus (Mammalia:Soricidae). Biol J. 1996; 57: 307–316.

[20] Hutterer R, Tranier M. The immigration of the Asian house shrew Suncus murinus into Africa and Madagascar In: Peters G, Hutterer R, edistors. Vertebrates in the Tropics. Germany: Museum Alexander Koenig, Bonn 1990; 309–319.

[21] Radimilahy C. Mahilaka, an eleventh to fourteenth century Islamic port: the first impact of urbanism on Madagascar In: Goodman SM, Patterson BD, editors. Natural change and human impact in Madagascar. Washington: Smithsonian Institution Press, DC 1997; 342–363.

[22] Marshall J, Quy DV, Gibson FL, Dung TC, Cavanaugh D. Ecology of plague in Vietnam. I. Role of Suncus murinus. Soc Exp Biol Med. 1967; 124(4): 1083–1086. 10.3181/00379727-124-31930 6024813

[23] Suntsov VV, Thi L, Huong V, Suntsova NI, Gratz NG. Plague foci in Viet Nam: zoological and parasitological aspects. Bull World Health Organ. 1997; 74(2): 117–123. PMC2486932 9185363

[24] Rasoamanana B, Rahalison L, Raharimanana C, Chanteau S. 1996. Comparison of Yersinia CIN agar and mouse inoculation assay for the diagnosis of plague. Trans R Soc Trop Med Hyg. 1996; 90: 651 9015505 10.1016/s0035-9203(96)90420-4

[25] Russell P, Nelson M, Whittington D, Green M, Eley SM, Titball RW. Laboratory diagnosis of plague. Br J Bio Sci. 1997; 54: 231–236 9624730

[26] Rasoamanana B, Leroy F, Boisier P, Rasolomaharo M, Buchy P, Carniel E, et al Field evaluation of an immunoglobulin G anti-F1 enzyme-linked immunosorbent assay for serodiagnosis of human plague in Madagascar. Clin Diagn Lab Immunol. 1997; 4(5): 587–591. 9302210 10.1128/cdli.4.5.587-591.1997 PMC170602

[27] Dromigny JA, Ralafiarisoa L, Raharimanana C, Randriananja N, Chanteau S. La sérologie anti-F1 chez la souris OF1, test complémentaire pour le diagnostic de la peste humaine. Arch Inst Pasteur Madagascar. 1998; 64: 18–20.

[28] Andrianaivoarimanana V, Telfer S, Rajerison M, Ranjalahy M, Andriamiarimanana F, Rahaingosoamamitiana C, et al Immune responses to plague infection in wild Rattus rattus, in Madagascar: A role in foci persistence? PLoS One. 2012; 7(6): 1–8. 10.1371/journal.pone.0038630 PMC3377696 22719908

[29] Chanteau S, Ratsitorahina M, Nato F. Development pneumonic and testing plague of a rapid diagnostic test for bubonic and. Lancet. 2003;361:211–6. doi: 10.1016/S0140-6736(03)12270-2 12547544 10.1016/S0140-6736(03)12270-2

[30] Morelli G, Song Y, Mazzoni CJ, Eppinger M, Roumagnac P, Wagner DM, et al Yersinia pestis genome sequencing identifies patterns of global phylogenetic diversity. Nat Publ Gr. 2010; 42(12): 1140–1143. 10.1038/ng.705 PMC2999892 21037571

[31] Burnham KP, Anderson DR. Multimodel inference: understanding AIC and BIC in model selection. Sociol Methods Res. 2004; 33: 261–304

[32] R. Core Team. R: A language and environment for statistical computing. R Fund. Stat. Comput. Vienna, Austria 2014.

[33] Haydon DT, Cleaveland S, Taylor LH, Laurenson MK. Identifying Reservoirs of Infection: A Conceptual and Practical Challenge. Emerg Infect Dis. 2002; 8(12): 1468–1473. doi: 10.3201/eid0812.010317 12498665 10.3201/eid0812.010317 PMC2738515

[34] Viana M, Mancy R, Biek R, Cleavand S, Cross PC, Lloyd-Smith JO, et al Assembling evidence for identifying reservoirs of infection. Trends Ecol Evol. 2014; 29(5): 270–279. doi: 10.1016/j.tree.2014.03.002 24726345 10.1016/j.tree.2014.03.002 PMC4007595

[35] Gage KL, Kosoy MY. Natural history of plague: perspectives from more than a century of research. Annu Rev Entomol. 2005; 50: 505–528. doi: 10.1146/annurev.ento.50.071803.130337 15471529 10.1146/annurev.ento.50.071803.130337

[36] Engelthaler DM, Hinnebusch BJ, Rittner CM, Gage KL. Quantitative competitive PCR as a technique for exploring flea-*Yersina pestis* dynamics. Am J Trop Med Hyg. 2000; 62: 552–560. 11289663 10.4269/ajtmh.2000.62.552

[37] Lorange EA, Race BL, Sebbane F, Hinnebusch BJ. Poor vector competence of fleas and the evolution of hypervirulence in *Yersinia pestis*. J Infect Dis. 2005; 191: 1907–1912. doi: 10.1086/429931 15871125 10.1086/429931

[38] Zhang Y, Dai X, Wang X, Maituohuti A, Cui Y, Rehemu A, et al Dynamics of Yersinia pestis and Its Antibody Response in Great Gerbils (Rhombomys opimus) by Subcutaneous Infection. Plos One. 2012; 7(10): 4–11. 10.1371/journal.pone.0046820 PMC3465280 23071647

[39] Williams JE, Cavanaugh DC. Chronic infections in laboratory rodents from inoculation of nonencapsulated plague bacilli (Yersinia pestis). Experientia. 1983; 39 (4): 408–409. 6832323 10.1007/BF01963151

[40] Du Y, Rosqvist R, Forsberg A. Role of fraction 1 antigen of *Yersinia pestis* in inhibition of phagocytosis, Infect. Immun. 2002; 70:1453–1460. doi: 10.1128/IAI.70.3.1453-1460.2002 11854232 10.1128/IAI.70.3.1453-1460.2002 PMC127752

[41] Sebbane F, Gardner D, Long D, Gowen BB, Hinnebusch BJ. Kinetics of disease progression and host response in a rat model of bubonic plague, Am. J. Pathol. 2005; 166:1427–1439. doi: 10.1016/S0002-9440(10)62360-7 15855643 10.1016/S0002-9440(10)62360-7 PMC1606397

[42] Davis S, Leirs H, Viljugrein H, Stenseth NC, De Bruyn L, Klassovskiy N, et al,. Empirical assessment of a threshold model for sylvatic plague. J R Soc Interface. 2007; 4: 649–657 doi: 10.1098/rsif.2006.0208 17254978 10.1098/rsif.2006.0208 PMC2373385

[43] Davis S, Trapman P, Leirs H, Begon M, Heesterbeek JAP. The abundance threshold for plague as a critical percolation phenomenon. Nature. 2008; 454: 634–637. doi: 10.1038/nature07053 18668107 10.1038/nature07053

[44] Williams JE, Hudson BW, Turner RW, Sulianti Saroso J, & Cavanaugh DC. Plague in Central Java, Indonesia. Bull World Health Organ. 1980; 58(3), 459–468. 6968252 PMC2395920

[45] Kreppel KS, Telfer S, Rajerison M, Morse A, Baylis M. Effect of temperature and relative humidity on the development times and survival of Synopsyllus fonquerniei and Xenopsylla cheopis, the flea vectors of plague in Madagascar. Parasit Vectors. 2016; 9(82). 10.1186/s13071-016-1366-z PMC4750303 26864070

