# Supplementary material for: The Asian house shrew Suncus murinus as a reservoir and source of human outbreaks of plague in Madagascar
Source: PLoS Negl Trop Dis. 2017 Nov 20;11(11):e0006072. doi: 10.1371/journal.pntd.0006072 (PMC5714386; doi:10.1371/journal.pntd.0006072)
Supplement: S1 Table — (PDF) [file pntd.0006072.s001.pdf]

S1 Table: Plague indicators during epidemics, post epidemics periods, and 15 years after the last human outbreak.

	August 1991 (first epidemic)*				1995 (second outbreak)							
Species	Animal trapped, no.	Flea index	*Y. pestis* positive no.	SP† (%)	Animal trapped, no.	Flea index	*Y. pestis* positive no.	SP† (%)				
M. musculus	0	NA	NA	NA	3	NA	0	NA				
R. norvegicus	2	NA	2	NA	15	NA	1	NA				
R. rattus	2	41	1	NA	3	NA	0	NA				
S. murinus	16	4	3	NA	94	5	1	NA				
	May 1997-May1999 (Epidemics)				Nov 1999 - Nov 2001 (Postepidemics)				Nov 2011 – Nov 2014 (Recent)			
Species	Animal trapped, no.	Flea index	*Y. pestis* positive no.	SP, %	Animal trapped, no.	Flea index	*Y. pestis* positive no.	SP, %	Animal trapped, no.	Flea index	*Y. pestis* positive no.	SP, %
M. musculus	34	0.3	0	13.3	24	0.1	0	20.8	37	0.3	0	11.1
R. norvegicus	87	8.6	3	16.0	76	7.4	0	0.0	174	4.3	1	2.3
R. rattus	41	3.1	0	4.9	16	2.8	0	0.0	17	2.7	0	0.0
S. murinus	654	2.2	5	9.1	181	2.2	0	7.8	192	1.2	0	11.7

*Source Laventure et al, 1998,

† Enzyme-Linked Immuno Sorbent Assay (ELISA) serological antibodies test in rodents has been developed in 1997.

SP : *Yersinia pestis* seroprevalence ; NA : not assessed ;

Flea index : the average number of fleas on a rat
