# Supplementary material for: The Asian house shrew Suncus murinus as a reservoir and source of human outbreaks of plague in Madagascar
Source: PLoS Negl Trop Dis. 2017 Nov 20;11(11):e0006072. doi: 10.1371/journal.pntd.0006072 (PMC5714386; doi:10.1371/journal.pntd.0006072)
Supplement: S2 Table — (PDF) [file pntd.0006072.s002.pdf]

S2 Table: Final Generalized linear models (GLM) models for analyses of small mammal and flea abundances

| Response variable | Type model | Final model | Test | p-value / ΔAIC |
|---|---|---|---|---|
| *S. murinus* abundance | Quasi-poisson | Time period | F-test | 0.0005 |
| | | Month | F-test | 0.007 |
| *R. norvegicus* abundance | Quasi-poisson | Time trend | F-test | 0.039 |
| Proportion *S. murinus* infested with fleas | Quasi-binomial | Time trend | F-test | 0.0007 |
| | | Month | F-test | <0.0001 |
| | | Site | F-test | 0.005 |
| Proportion *R. norvegicus* infested with fleas | Quasi-binomial | No significant variables | | |
| Flea index on *S. murinus* | Negative binomial | Time trend | AIC | 3.45 |
| | | Month | | 16.19 |
| | | Site | | 8.07 |
| Flea index on *R. norvegicus* | Negative binomial | Month | AIC | 3.42 |

AIC : Akaike Information Criterion
