# Supplementary material for: The Asian house shrew Suncus murinus as a reservoir and source of human outbreaks of plague in Madagascar
Source: PLoS Negl Trop Dis. 2017 Nov 20;11(11):e0006072. doi: 10.1371/journal.pntd.0006072 (PMC5714386; doi:10.1371/journal.pntd.0006072)
Supplement: S3 Table — (PDF) [file pntd.0006072.s003.pdf]

S3 Table: Coefficients from final GLM models for small mammal and flea abundance

	Estimate	SE	t-value	p-value
S. murinus abundance				
Intercept	3.367	0.192	17.48	<0.001
Post-epidemic	-0.573	0.172	-3.33	0.002
Recent	-0.808	0.203	-3.97	<0.001
May	0.540	0.232	2.32	0.027
August	0.017	0.271	0.06	0.950
November	0.026	0.245	0.10	0.917
R. norvegicus abundance				
Intercept	1.615	0.201	8.03	<0.001
Time	0.044	0.020	2.22	0.033
Proportion S. murinus infested with fleas				
Intercept	-1.370	0.379	-3.62	0.001
Time	-0.082	0.022	-3.69	<0.001
May	1.281	0.393	3.26	0.003
August	3.032	0.554	5.47	<0.001
November	1.782	0.417	4.27	<0.001
Tsararano	0.700	0.227	3.09	0.004
Flea index on S. murinus				
Intercept	-0.775	0.335	-2.31	0.021
Time	-0.047	0.019	-2.53	0.011
May	0.829	0.364	2.28	0.022
August	2.184	0.432	5.06	<0.001
November	1.444	0.363	3.98	<0.001
Tsararano	0.709	0.202	3.50	<0.001
Flea index on R. norvegicus				
Intercept	1.964	0.281	6.99	<0.001
May	-0.363	0.324	-1.12	0.263
August	0.658	0.404	1.63	0.104
November	-0.028	0.322	-0.09	0.930
